# Supplementary material for: Environmental motion presented ahead of self-motion modulates heading direction estimation
Source: PLoS Comput Biol. 2025 Oct 9;21(10):e1013571. doi: 10.1371/journal.pcbi.1013571 (PMC12527208; doi:10.1371/journal.pcbi.1013571)
Supplement: S1 Supporting Information — (PDF) [file pcbi.1013571.s001.pdf]

S1 Supporting Information

Supplemental Methods

To ensure precise temporal alignment between the visual and vestibular stimuli, we implemented a synchronization procedure. The results of this procedure are shown in **Fig A**, which displays the commanded and measured motion profiles before and after delay compensation.

Both the input command to the motion platform and the resulting physical displacement are represented in terms of position. While the input was specified in millimeters, the actual platform movement was monitored using encoder data expressed in hardware-specific counts. These encoder counts, though not directly in millimeters, are linearly proportional to the motor shaft rotation and thus to the crank arm angle driving the platform's translation. Because of the fixed mechanical linkage, this proportionality ensures that encoder counts reliably track changes in linear displacement, allowing us to compare commanded and actual motion trajectories with consistent temporal precision. The precise conversion factor between encoder counts and millimeters was handled by the platform's control system and was not required for the delay analysis, which relied on relative timing and linearity between command and response trajectories. By comparing the commanded trajectory (input) with the measured encoder-derived trajectory (response), we observed a consistent delay in the platform's physical response relative to the command. Specifically, the response onset lagged behind the input by approximately 5 frames, or 83 ms at a 60 Hz sampling rate.

To correct for this measured delay, the onset of the visual stimulus was shifted forward by 5 frames (83 ms), thereby aligning its perceived motion onset with the actual movement of the platform. After compensation, the alignment between the commanded and measured profiles improved significantly, demonstrating that the visual and vestibular stimuli were synchronized in both time and trajectory shape.

As an additional qualitative verification, a moving dot mimicking the platform motion was displayed on the screen and aligned with a physical reference marker mounted on the platform. From the participant's viewpoint, the moving dot and the physical marker moved in unison, confirming perceptual synchrony between visual and vestibular motion cues.

Although our calibration procedure cannot remove all the discrepancy between input and response due to the inherent noise in the system, the difference between input and output speed profiles after our calibration procedure falls well within the known temporal window of integration for visual and vestibular signals. For instance, Rodriguez and Crane [S1] reported that visual motion influences inertial heading perception when intermodal timing differences are within 250 ms, indicating that our system remains well within the perceptual threshold for the natural multisensory integration.

Supplemental References

S1. Rodriguez R, Crane BT. Effect of timing delay between visual and vestibular stimuli on heading perception. J Neurophysiol. 2021;126(1):304-12.

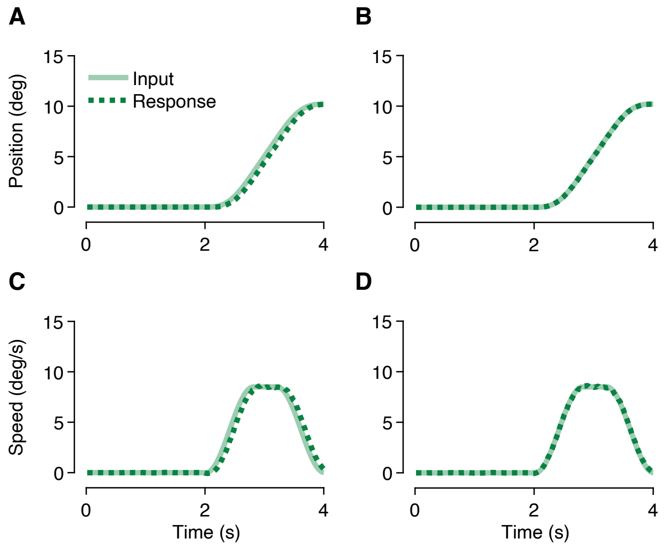

Fig A. Motion platform synchronization before and after delay compensation. (**A**,**B**) Position profiles of the commanded trajectory (light green) and measured response (dark green) before (**A**) and after (**B**) applying an 83 ms delay compensation. (**C**,**D**) Corresponding speed profiles by differentiating the position data and smoothing with a Savitzky-Golay filter (window size: 450 ms).

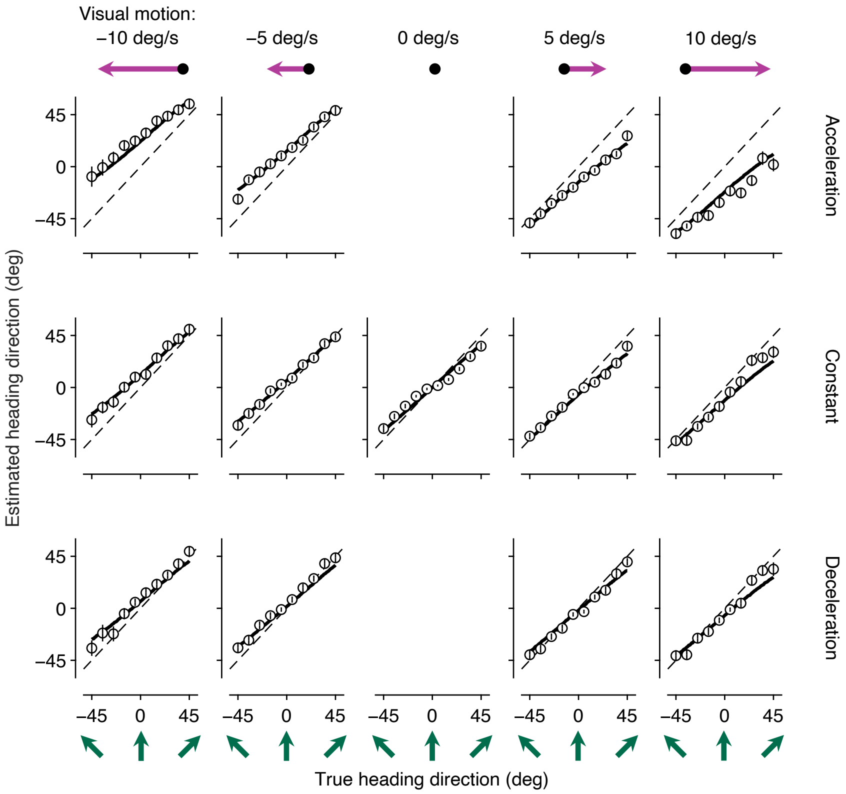

Fig B. Group average. Same as in **Fig 2** but for the group average. Error bars represent the standard error of the mean.

Table A. Parameter estimates. Numbers indicate the mean (SEM) and [median] across observers.

	CCI	Int	Seg	Cov	Fix	Heu	WTA	MCI
σ_{self}	7.71 (1.11) [6.73]	9.68 (1.68) [7.46]	8.66 (1.58) [6.04]	9.21 (1.58) [7.84]	-	6.91 (1.04) [5.87]	7.27 (1.12) [6.29]	7.57 (1.31) [6.29]
σ_{env}	7.14 (1.17) [6.25]	6.53 (1.14) [4.88]	7.65 (1.00) [6.37]	6.93 (0.56) [6.64]	-	5.08 (0.44) [4.94]	6.21 (0.77) [5.52]	-
σ_{vest}	4.79 (0.80) [3.62]	3.53 (0.61) [2.78]	4.39 (0.88) [3.36]	5.36 (1.01) [3.88]	6.33 (1.48) [3.72]	5.85 (1.48) [3.42]	4.09 (0.58) [3.41]	5.43 (1.56) [3.10]
w_{vis}	0.238 (0.021) [0.205]	0.575 (0.015) [0.600]	0.197 (0.034) [0.168]	0.166 (0.032) [0.141]	0.303 (0.039) [0.288]	0.321 (0.028) [0.319]	0.267 (0.037) [0.243]	0.338 (0.044) [0.366]
p_{constant}	0.305 (0.047) [0.267]	-	-	-	-	-	0.321 (0.079) [0.289]	-
ρ	-	-	-	0.486 (0.057) [0.516]	-	-	-	-
α_{vest}	-	-	-	-	0.449 (0.058) [0.453]	-	-	-
α_{mom}	-	-	-	-	0.126 (0.022) [0.096]	-	-	-
α_{cont}	-	-	-	-	0.183 (0.033) [0.193]	-	-	-
p_{common}	-	-	-	-	-	-	-	0.456 (0.068) [0.498]

Table B. The CCI model parameter estimates and R^2 for individual observers.

	σ_{self}	σ_{env}	σ_{vest}	w_{vis}	p_{constant}	R^2
Observer 1	11.31	5.11	3.15	0.179	0.102	0.738
Observer 2	13.10	7.34	5.70	0.286	0.692	0.732
Observer 3	5.64	6.08	4.76	0.210	0.284	0.656
Observer 4	6.49	7.28	8.81	0.167	0.215	0.613
Observer 5	6.96	6.42	3.96	0.278	0.270	0.668
Observer 6	4.47	3.92	5.42	0.185	0.247	0.762
Observer 7	9.46	12.45	3.24	0.313	0.412	0.742
Observer 8	3.13	3.42	1.51	0.153	0.263	0.841
Observer 9	15.81	7.17	6.31	0.300	0.552	0.674
Observer 10	2.62	3.87	2.11	0.164	0.234	0.739
Observer 11	11.03	5.91	2.91	0.407	0.419	0.724
Observer 12	3.33	19.99	3.08	0.331	0.060	0.621
Observer 13	4.24	3.37	3.28	0.155	0.411	0.788
Observer 14	10.35	7.67	12.80	0.199	0.111	0.702
