# Supplementary material for: Environmental motion presented ahead of self-motion modulates heading direction estimation
Source: PLoS Comput Biol. 2025 Oct 9;21(10):e1013571. doi: 10.1371/journal.pcbi.1013571 (PMC12527208; doi:10.1371/journal.pcbi.1013571)
Supplement: Attachment — Submitted filename: ResponseToReviewer_R1.pdf [file pcbi.1013571.s006.pdf]

Dear Dr. Bays and Dr. Graham,

Thank you for considering our manuscript for publication in PLOS Computational Biology. We appreciate the opportunity to revise and resubmit the manuscript and believe that it has been substantially improved thanks to the input by reviewers.

Based on the constructive comments provided by the reviewers, we substantially revised the manuscript. The key changes include:

- Confirmation of visual & vestibular stimuli synchrony,
- Presentations of individual-level results,
- Comparisons with alternative models, and
- Text edits to Methods, Results and Discussion for clarity.

The reviewers' comments are fully quoted below; our responses follow each comment with right indents. All non-trivial changes to the manuscript are highlighted in blue and also reproduced and/or summarized below.

Sincerely,

Oh-Sang Kwon, PhD
Associate Professor
Department of Biomedical Engineering
Ulsan National Institute of Science and Technology
oskwon@unist.ac.kr

Reviewer #1

This paper examines how visual motion before a self-motion influences heading perception. Building on past work that typically focuses only on concurrent visual–vestibular inputs, the authors present a paradigm where participants are moved on a platform while viewing visual stimuli that vary in speed and velocity profile prior to the movement. Their main finding is that these pre-movement stimuli bias subsequent heading estimates. They propose a Bayesian causal-inference model that combines (1) vestibular information, (2) concurrent visual motion, and (3) contextual (pre-movement) visual signals. Their conclusion is that the brain integrates cues about self-motion across time.

Main criticisms;
1. Inadequate presentation of individual-participant data
Group-level Averaging vs. Individual Patterns. The paper relies heavily on group-averaged results (e.g., Figure 2), but the authors' claims emphasize that heading perception and causal inference are implemented in individual brains. If the claims are indeed about individuals, more subject-level detail is essential to confirm that effects hold systematically across participants (e.g., violin plots or per-subject fits).
Model Fits and Variability. The manuscript would benefit from showing how well the Bayesian model explains each individual's data (variance explained, parameter values, etc.). The current group-level approach may mask considerable individual differences, and it raises concerns about underpowered inferences if the main statistical tests are done at the group level.

We thank the reviewer for the helpful comments. We agree that only showing group-averaged results was not sufficient to make claims about perceptual processing of the individuals. In response, we revised Fig. 2 (now **Fig. 3** in the revised manuscript) to have not only group-level results (**Fig. 3B**) but also individual fits (**Fig. 3C**). In addition, most of the additional figures we plotted in the revised manuscript (**Figs. 2, 4A, 4B, 4D** and **5B**) contain individual-level data. All results conform to the group-level analysis, further strengthening the key findings in our study.

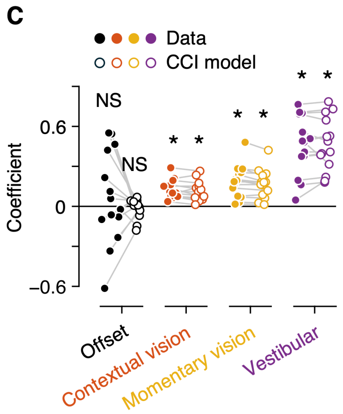

Fig. 3. (**C**) Linear regression coefficients for human (filled dots) and the CCI model (open dots) observers. $^*P < 0.05$, NS $P > 0.05$.

To show how well our model explains each individual's data, we now report R^2 (mean ± SEM = 0.714 ± 0.017; **Line 183**), as suggested by the reviewer. We also quantitatively compare the goodness-of-fit with other alternative models using AIC (**Lines 207, 214, 222** and **245**) and show the results on the individual level (**Fig. 4A**). Lastly, we report parameter values for individual observers (**Table S2**), as suggested by the reviewer.

Table S2. The CCI model parameter estimates and R^2 for individual observers.

	σ_{self}	σ_{env}	σ_{vest}	w_{vis}	p_{constant}	R^2
Observer 1	11.31	5.11	3.15	0.179	0.102	0.738
Observer 2	13.10	7.34	5.70	0.286	0.692	0.732
Observer 3	5.64	6.08	4.76	0.210	0.284	0.656
Observer 4	6.49	7.28	8.81	0.167	0.215	0.613
Observer 5	6.96	6.42	3.96	0.278	0.270	0.668
Observer 6	4.47	3.92	5.42	0.185	0.247	0.762
Observer 7	9.46	12.45	3.24	0.313	0.412	0.742
Observer 8	3.13	3.42	1.51	0.153	0.263	0.841
Observer 9	15.81	7.17	6.31	0.300	0.552	0.674
Observer 10	2.62	3.87	2.11	0.164	0.234	0.739
Observer 11	11.03	5.91	2.91	0.407	0.419	0.724
Observer 12	3.33	19.99	3.08	0.331	0.060	0.621
Observer 13	4.24	3.37	3.28	0.155	0.411	0.788
Observer 14	10.35	7.67	12.80	0.199	0.111	0.702

Lastly, while carefully examining individual data according to the reviewer's suggestion, we found that one observer reported only ±30° across all trials, as if this observer performed a 2AFC discrimination task as to whether heading direction was clockwise or counterclockwise relative to vertically upward (**Revision Fig. 1**). We excluded this observer's data from analysis. We sincerely appreciate the reviewer for pointing out the importance of examining individual observers' data. We described this exclusion in the revised manuscript (**Line 344**).

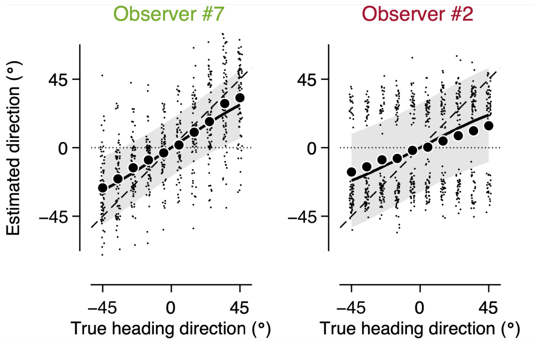

Revision Fig. 1. Unlike the other observers with unimodal response distributions (e.g., Observer #7), Observer #2 always reported ±30° across all trials. The bimodal peak and variance of compound response distribution were the same across different heading directions, from which we suspect that this observer misunderstood the task instruction and reported whether the heading direction was clockwise or counterclockwise relative to vertical.

[Line 344] We excluded one observer's data from analysis, as this observer always reported ±30° throughout the experiment, presumably misunderstanding the task instruction.

2. Overprocessed data

The data presented in Fig 2. appears to have averaged over the 10 different motion directions that were presented. Why are biases not shown as a function of that direction?

In the revised manuscript, we now present the results for all unique stimulus conditions, plotting the estimated heading direction as a function of true heading directions for five visual motion velocities and three contextual visual motion conditions. We observed that the heading estimates were systematically biased in the direction opposite to the visual motion stimuli, with the magnitude of the bias depending on both visual motion speed and condition. This was true in both individual observers (**Fig. 2**) and the group average (**Fig. S2**). We also observed that the heading estimates were biased to the cardinal axes, a pattern that is well-established in literature (Girshick et al., 2013; Wei & Stocker, 2015). We did not explicitly model the cardinal bias in any of our models, although the zero-mean prior would partly capture it. That said, we would like to point out that our model still provides a good fit to the data (**Figs. 2** and **S2**) even without the explicit modeling of the cardinal bias. All of our observations are now included in the revised manuscript (**Line 93**).

[Line 93] Observers' heading estimation behavior exhibited three characteristic features (**Fig. 2**; see also **Fig. S2** for the group average). First, heading estimates were systematically biased in the direction opposite to the visual motion stimuli. When visual motion stimuli moved leftward, heading estimates were biased clockwise, and when visual motion stimuli moved rightward, heading estimates were biased counterclockwise. Notably, observers did not fully compensate for the motion in the environment, leading to a biased heading estimate even when the environmental motion remained constant before and during self-motion. Second, for each visual motion condition, the strength of heading biases depended on the speed of visual motion stimuli, such that heading biases were more pronounced with faster visual motion stimuli. Third, the strength of heading biases depended on visual motion stimuli presented ahead of self-motion. Specifically, heading biases were more pronounced in Constant condition than in Deceleration condition, and even more so in Acceleration condition.

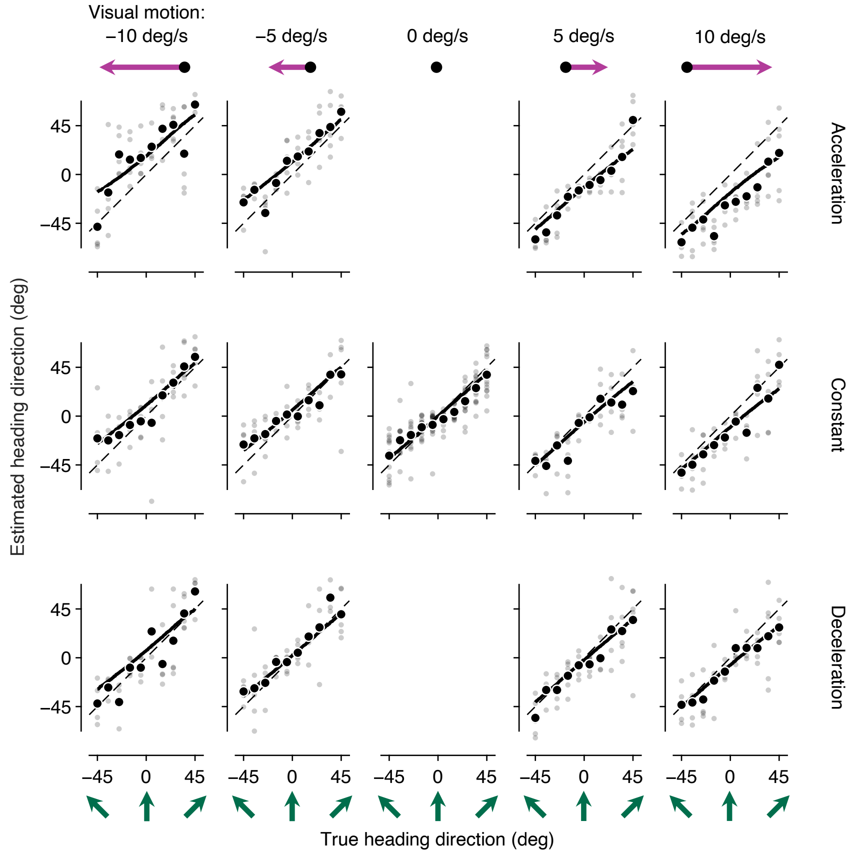

Fig. 2. Overview of the heading estimation behavior. A representative human observer's heading estimates for every trial (small semi-transparent dots) and their averages for each heading direction (large filled circles) for each heading direction are shown for five visual motion velocities (plotted in each column) and three visual motion conditions (plotted in each row) along with the prediction of the Contextual Causal Inference model (solid lines). Heading estimates deviate on average from the unity line (diagonal dashed line) depending on visual motion velocities and visual motion conditions.

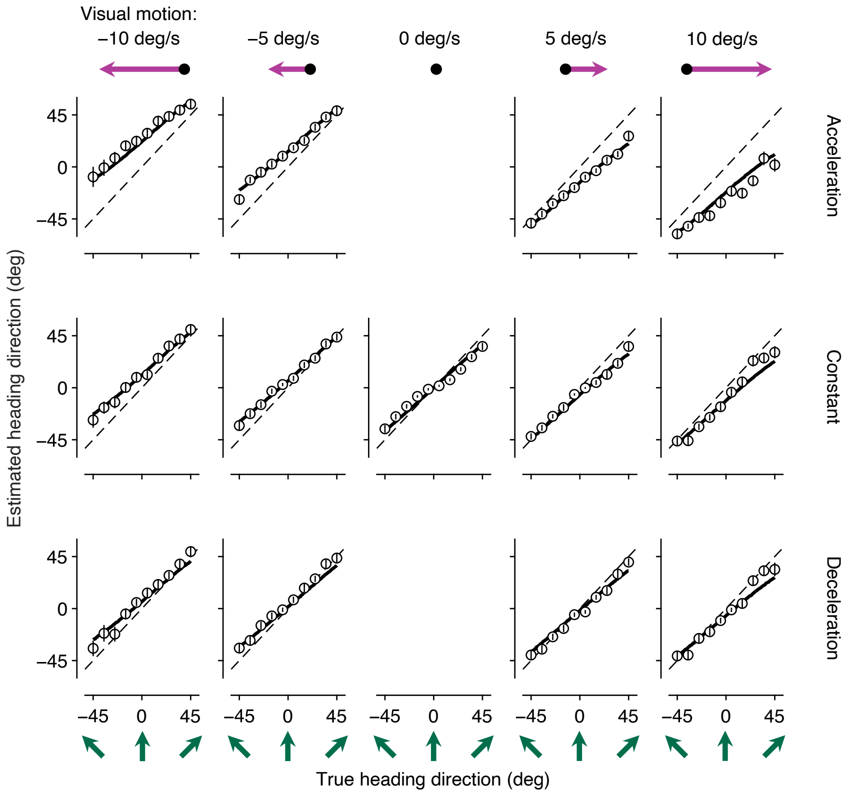

Fig. S2. Group average. Same as in **Fig. 2** but for the group average. Error bars represent the standard error of the mean.

3. Clarity on "Optimal" Estimates and the Underlying Loss Function
Lines 114 and 120: Confusion About "Optimal." The text uses "optimal estimate" in contexts that invoke both mean-based (L2 loss) and MAP (L0 or similar) solutions, but these concepts have distinct mathematical meanings. For instance, line 114 refers to a model-averaging approach (optimal for L2 loss), yet line 120 seems to imply a MAP approach (optimal under a different loss function).
Line 134: "Optimal Solution That Maximizes Posterior Probability." Here again, the authors equate maximizing the posterior with an "optimal solution," but this is only correct for L0 loss. While these in accuracies don't appear consequential for this particular manuscript since L0, L1, and L2 all imply the same for the Gaussian posteriors assumed here, the manuscript's terminology should be made more precise.

We thank the reviewer for raising this important point. We now edited the manuscript such that it consistently uses the objective of minimizing the expected squared error (i.e., L2 loss), following previous studies that showed that humans use L2 loss in behavioral tasks (Körding & Wolpert, 2004; Jazayeri & Shadlen, 2010). We also vastly edited the Methods to clarify how we derive an optimal estimate under the assumption of L2 loss (starting from **Line 456**). Below are edits to the lines the reviewer specifically mentioned.

[Line 156] We found that the optimal estimate that minimizes the posterior expected loss in each scenario is given by [...]

[Line 171] Instead, these computations, including the subtraction, naturally emerged as an optimal solution that minimizes the posterior expected loss (**Methods**).

[Line 456] With this generative model in mind, the observer infers the direction of self-motion from noisy and ambiguous sensory signals. To do so, the observer first constructs the posterior probability distribution over self-motion, $p(s_{\text{self}}|x_{\text{vest}}, x_{\text{vis}}, x_{\text{vis0}})$, which represents the observer's belief about self-motion, s_{self}, after receiving the sensory signals, x_{vest}, x_{vis} and x_{vis0}. From this posterior, the observer computes a single estimate, $\hat{s}_{\text{self}}$, by considering a loss function, $\ell(\hat{s}_{\text{self}}, s_{\text{self}})$, which quantifies the cost of erroneously estimating s_{self} as $\hat{s}_{\text{self}}$. The Bayesian estimate is the one that minimizes the posterior expected loss, which is the expected value of the loss function under the posterior distribution over s_{self}.

$$\hat{s}_{\text{self}} = \underset{\hat{s}_{\text{self}}}{\operatorname{argmin}} \int \ell(\hat{s}_{\text{self}}, s_{\text{self}}) p(s_{\text{self}}|x_{\text{vest}}, x_{\text{vis}}, x_{\text{vis0}}) ds_{\text{self}} \qquad (7)$$

Since the generative model depends on the latent causal structure, C, of the motion in the environment, inference about self-motion must also depend on C. However, the observer does not have access to the true causal structure. We therefore rewrite the posterior of self-motion as a marginalization over all possible causal structures:

$$p(s_{\text{self}}|x_{\text{vest}}, x_{\text{vis}}, x_{\text{vis0}}) = \sum_{C} p(s_{\text{self}}|x_{\text{vest}}, x_{\text{vis}}, x_{\text{vis0}}, C) p(C|x_{\text{vest}}, x_{\text{vis}}, x_{\text{vis0}}) \qquad (8)$$

Assuming a squared error loss, $\ell(\hat{s}_{\text{self}}, s_{\text{self}}) = (\hat{s}_{\text{self}} - s_{\text{self}})^2$ [91,92], the optimal estimate becomes the mean of the posterior. Substituting the marginalized posterior into the estimator, we obtain:

$$\hat{s}_{\text{self}} = \int s_{\text{self}}\, p(s_{\text{self}}|x_{\text{vest}}, x_{\text{vis}}, x_{\text{vis0}}) ds_{\text{self}} = \sum_{C} p(C|x_{\text{vest}}, x_{\text{vis}}, x_{\text{vis0}}) \int s_{\text{self}}\, p(s_{\text{self}}|x_{\text{vest}}, x_{\text{vis}}, x_{\text{vis0}}, C) ds_{\text{self}} \qquad (9)$$

Thus, the optimal heading estimate becomes a combination of two optimal heading estimates assuming each causal structure C, weighted by its posterior probability (i.e., model averaging [93]). To compute the optimal heading estimate, the observer has to infer whether environmental motion has remained constant, and simultaneously compute the optimal heading estimates assuming that environmental motion has remained constant or has changed.

4. Bayesian Model vs. Regression Models: Significance of Coefficients
3a) The paper appears to use two different kinds of models, a regression model, and a Bayesian model. While the equations are provided for the latter, the equations for the former would be helpful.

In response, we included the equations for the linear regression analysis (**Line 420**).

[Line 420] To quantitatively assess contributions of each source of information, we fit a linear regression model with the observers' heading estimate as a dependent variable and the predicted heading bias for each source of information as independent variables:

$$\hat{s}_{\text{self}} = \beta_0 + \beta_1 s_{\text{self}} + \beta_2(s_{\text{self}} - s_{\text{env}}) + \beta_3(s_{\text{self}} - s_{\text{env}} + s_{\text{env0}}) \quad (6)$$

where s_{self} represents self-motion and s_{env} and s_{env0} represent environmental motion before and during self-motion, respectively (see also below).

3b) I assume the Bayesian model was fit to individual observers but virtually no details were given on the results of this fitting: how many parameters were fit, what were their values, how good was the fit on an individual subject level, etc

Yes, the model was fit to individual observers' data, which is now more clearly stated in the revised manuscript (**Line 639**). In response to the reviewer's comment, we added detailed explanations of model fitting ("**Model fitting**" in **Methods**; **Line 617**), including how many parameters were fit for each model. We also reported parameter values for all models (**Tables S1** and **S2**). Lastly, we reported individual fit metrics, such as R^2 (**Line 183**) and AIC (**Fig. 4A**).

[Line 639] We used **Eq. 49** to minimize the negative log posterior probability of model parameters given all heading estimates measured psychophysically for each observer.

Table S1. Parameter estimates. Numbers indicate the mean (SEM) and [median] across observers.

	CCI	Int	Seg	Cov	Fix	Heu	WTA	MCI
σ_{self}	7.71 (1.11) [6.73]	9.68 (1.68) [7.46]	8.66 (1.58) [6.04]	9.21 (1.58) [7.84]	-	6.91 (1.04) [5.87]	7.27 (1.12) [6.29]	7.57 (1.31) [6.29]
σ_{env}	7.14 (1.17) [6.25]	6.53 (1.14) [4.88]	7.65 (1.00) [6.37]	6.93 (0.56) [6.64]	-	5.08 (0.44) [4.94]	6.21 (0.77) [5.52]	-
σ_{vest}	4.79 (0.80) [3.62]	3.53 (0.61) [2.78]	4.39 (0.88) [3.36]	5.36 (1.01) [3.88]	6.33 (1.48) [3.72]	5.85 (1.48) [3.42]	4.09 (0.58) [3.41]	5.43 (1.56) [3.10]
w_{vis}	0.238 (0.021) [0.205]	0.575 (0.015) [0.600]	0.197 (0.034) [0.168]	0.166 (0.032) [0.141]	0.303 (0.039) [0.288]	0.321 (0.028) [0.319]	0.267 (0.037) [0.243]	0.338 (0.044) [0.366]
p_{constant}	0.305 (0.047) [0.267]	-	-	-	-	-	0.321 (0.079) [0.289]	-
ρ	-	-	-	0.486 (0.057) [0.516]	-	-	-	-
α_{vest}	-	-	-	-	0.449 (0.058) [0.453]	-	-	-
α_{cont}	-	-	-	-	0.183 (0.033) [0.193]	-	-	-
α_{mom}	-	-	-	-	0.126 (0.022) [0.096]	-	-	-
p_{common}	-	-	-	-	-	-	-	0.456 (0.068) [0.498]

3c) The manuscript reports statistical significance ("stars") for regression coefficients associated with data but it's unclear whether they apply to the model fits (Fig 2b).

We now included markers (i.e., NS or *) for both human and model observers in **Fig. 3C**. We also report statistics in the text (**Line 186**).

[Line 186] All three coefficients were significant (contextual vision: $t_{13} = 5.716$, $P < 0.001$; momentary vision: $t_{13} = 5.848$, $P < 0.001$; vestibular: $t_{13} = 8.633$, $P < 0.001$; **Fig 3C**, open dots) [...]

3d) Relatedly, the authors discuss linear regression coefficients for different "strategies" (e.g., relying purely on vestibular information vs. adding contextual vision), but it is unclear how these regression values map onto the actual Bayesian model parameters.

The mapping from the causal inference model parameters and the linear regression coefficients is highly nonlinear, because the causal inference model performs a nonlinear integration of sensory cues (i.e., predictors in the linear regression analysis). We can still forcefully derive the mapping specific for the experimental parameters in our study, but it might mislead readers into thinking the causal inference model is a linear model. Instead, we directly plot the comparison between the linear regression coefficients obtained from human and model observers (**Fig. 4B**).

5. Evidence for Causal Inference vs. Forced Integration
Although the authors frame the Bayesian model as implementing causal inference (i.e., deciding whether environmental motion remains constant or changes over time), it is not entirely clear that there is strong behavioral evidence for a true "inference" process as opposed to simpler forced-integration strategies. Additional model comparisons would be valuable. Demonstrating that a forced-integration or fixed-weights model clearly performs worse than their "causal" approach would strengthen the central causal-inference claim. Similarly, what qualitative signatures would be expected from these alternative models in terms of the data obtained from this experiment?

We thank the reviewer for the valuable comments. In the revised manuscript, we performed two lines of analyses to address the reviewer's comments: 1) model fitting and model comparison of alternative models that have been considered in the previous studies, and 2) demonstration of a qualitative pattern of causal inference (and not cue combination with a fixed weight).

We built an Integration (Int) model and Fixed Weight (Fix) model, as suggested by the reviewer. The Int model is a special case of the causal inference model that always assumes constant environmental motion. The Fix model is rather a descriptive model that produces a heading estimate as a linear integration of sensory signals with fixed weights.

[Line 526]

$$\hat{s}_{\mathrm{self}} = \frac{\dfrac{x_{\mathrm{vest}}}{\sigma_{\mathrm{vest}}^2} + \dfrac{\mu_{\mathrm{K}}}{\sigma_{\mathrm{K}}^2} + \dfrac{0}{\sigma_{\mathrm{self}}^2}}{\dfrac{1}{\sigma_{\mathrm{vest}}^2} + \dfrac{1}{\sigma_{\mathrm{K}}^2} + \dfrac{1}{\sigma_{\mathrm{self}}^2}} \qquad (23)$$

where

$$\mu_{\mathrm{K}} = x_{\mathrm{vis}} - \frac{\sigma_{\mathrm{env}}^2}{\sigma_{\mathrm{env}}^2 + \sigma_{\mathrm{vis0}}^2} x_{\mathrm{vis0}} \qquad (24)$$

$$\sigma_{\mathrm{K}}^2 = \sigma_{\mathrm{vis}}^2 + \frac{\sigma_{\mathrm{env}}^2 \sigma_{\mathrm{vis0}}^2}{\sigma_{\mathrm{env}}^2 + \sigma_{\mathrm{vis0}}^2} \qquad (25)$$

[Line 566]

$$\hat{s}_{\mathrm{self}} = \alpha_{\mathrm{vest}} x_{\mathrm{vest}} + \alpha_{\mathrm{mom}} x_{\mathrm{vis}} + \alpha_{\mathrm{cont}} (x_{\mathrm{vis}} - x_{\mathrm{vis0}}) \qquad (35)$$

We also considered other alternative models such as conventional causal inference model in literature (Momentary Causal Inference model; MCI) that does not consider environmental motion presented ahead of self-motion, Segregation (Seg) model that always assumes environmental motion has changed before and during self-motion, and Covariance (Cov) model that assumes environmental motion before and during self-motion covaries and therefore performs a linear integration of sensory signals.

We found that unlike the Contextual Causal Inference model, alternative models that does not consider all available sensory information (i.e., vestibular, momentary vision, contextual vision) cannot reproduce the qualitative patterns of the heading bias (**Fig. 4B**) with linear regression coefficients significantly different from those of human observers (**Fig. 4C**). We quantitatively compared goodness-of-fit of models using AIC (**Fig. 4A**). All results confirm that the Contextual Causal Inference model outperforms other simpler models.

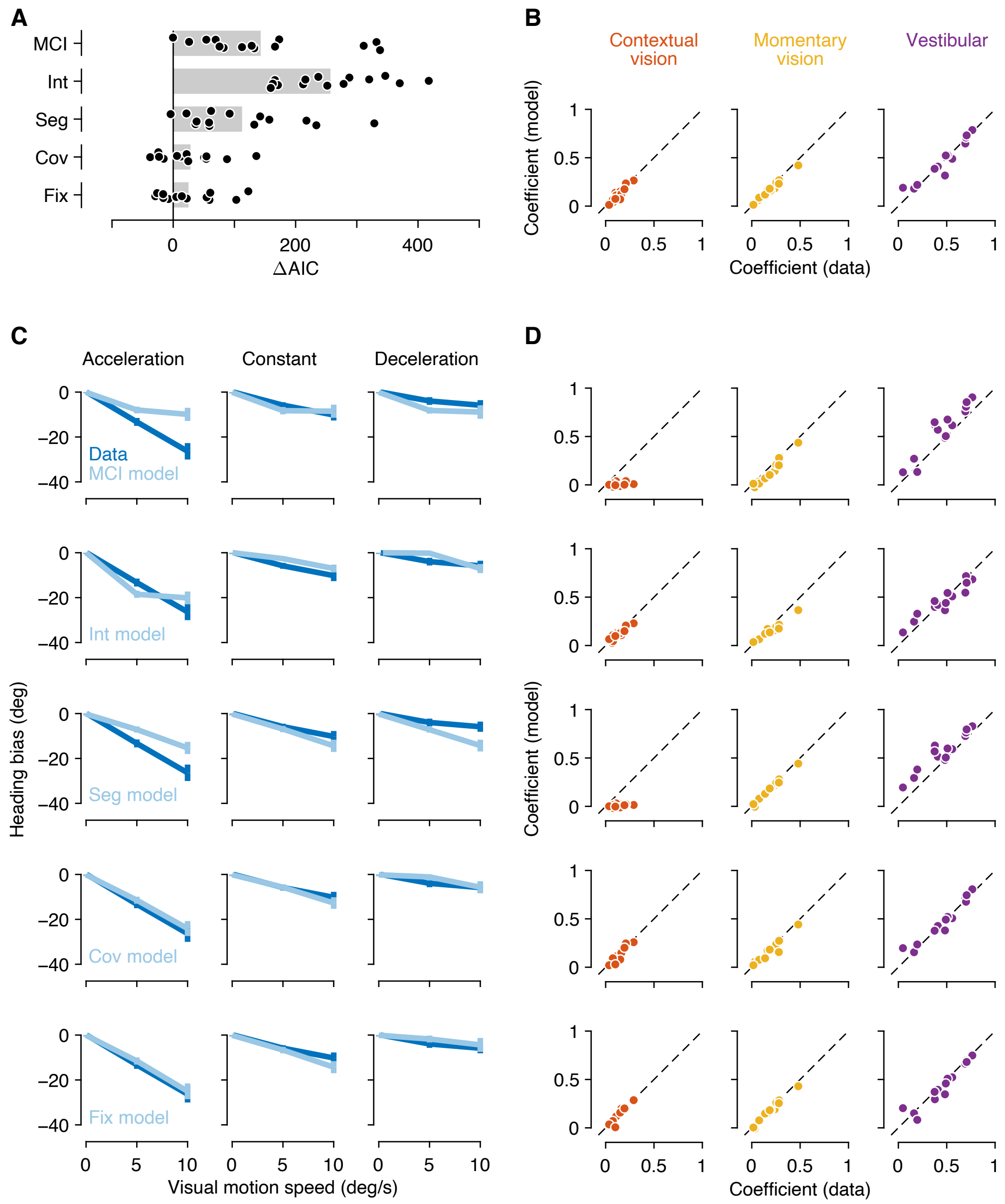

Fig. 4. Alternative models. (**A**) Comparison of goodness-of-fit among observer models. Small dots represent individual observers' difference in Akaike information criterion (AIC) values between each model and the CCI model, and gray bars represent the average. Positive values indicate a worse fit

than the CCI model. (**B**) Comparison of linear regression coefficients between human and CCI model observers. (**C**) Same as in **Fig. 3A** but for alternative models. (**D**) Same as in **B** but for alternative models.

We also asked whether observers performed a linear integration of sensory signals with fixed weights, without inferring the probability of each scenario. Our Causal Inference model indicates that observers evaluate the degree to which distinct sensory cues are different from one another (with respect to the sensory uncertainty associated with each cue) and use it as a weight on the heading estimates associated with the corresponding scenario. Therefore, the observers would use a larger weight on heading estimates associated with the constant environmental motion (i.e., $C = 1$) scenario when the visual motion stimuli presented before and during self-motion are actually the same, and use a smaller weight when they are actually different. To test this, we allowed the weight on the heading estimate associated with the $C = 1$ scenario to vary for trials where the environmental motion was actually same or different before and during self-motion. We found that, consistent with the causal inference prediction, human observers adaptively adjusted their weights according to whether the environmental motion was constant (**Fig. 5**).

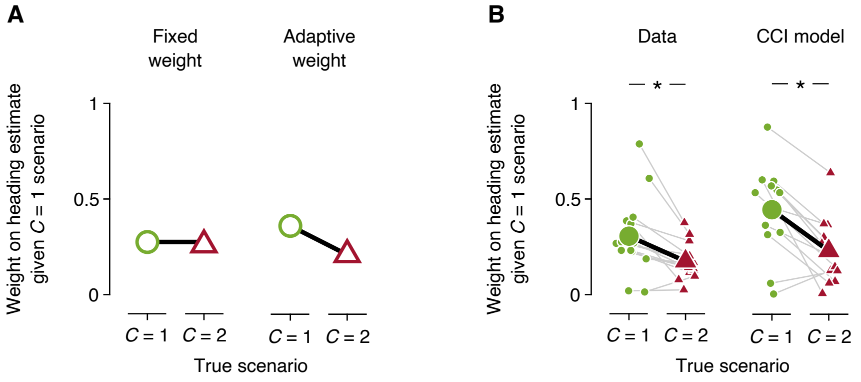

Fig. 5. Adaptive weight as a signature of causal inference. (**A**) Prediction about weights on the heading estimate associated with constant motion in the environment. For the linear sensory integration with fixed weights (left), the weights would be the same. For the nonlinear integration with adaptive weights (right), the weights would be larger when the environmental motion was constant (i.e., $C = 1$). (**B**) Weights on the heading estimate associated with constant motion in the environment for human (left) and the CCI model (right) observers. Small markers connected by gray lines represent weights for individual observers, and big markers connected by a black line represent the corresponding averages. $^*P < 0.05$.

Finally, we acknowledge that an adaptive weight does not necessarily mean observers performed causal inference in a Bayes-optimal manner. It is also possible that observers did it suboptimally. To test for this possibility, we fit a Heuristic model which compares cue disparities without taking into account the cue reliability and a Winner-Take-All model that performs a model selection. We found that both models provide a significantly worse fit than our causal inference model for all observers' data.

[Line 242] The results that the observers performed a contextual causal inference does not necessarily mean they did it optimally. To test this, we fit two variants of causal inference model: a Heuristic model that performs the causal inference without taking into account sensory uncertainty and a Winner-Take-All model that commits entirely to the more probable interpretation without taking into account the less probable one. Both the Heuristic model (64.7 [39.5 93.1]) and the Winner-Take-All model (39.7 [20.3 60.5]) showed a significantly worse fit than the CCI model.

6. Undiscussed Parameters and Fits
How many free parameters does the Bayesian model have, and how are they estimated? The paper mentions a Monte Carlo procedure but leaves open questions about prior assumptions, parameter fitting details (e.g., prior variance, Weber fraction, probability of the environment being static), and how the authors handled the stochastic likelihood of each trial (lines 351–353). An expanded methods section would help solidify confidence in the modeling approach.

Following the reviewer's suggestion, we vastly edited **Methods** with more details in model specifications (starting from **Line 426**) and model fitting (starting from **Line 617**). To answer the reviewer's question, we used Monte Carlo methods to compute the response distribution, $p(\hat{s}_{\text{self}}|s_{\text{self}}, s_{\text{env}}, s_{\text{env0}})$, which is a common practice in literature (e.g., Zhu et al., 2024). We assumed that observers' heading estimates are independent across trials and calculated the joint log likelihood of all trial data as the sum of log likelihood for each trial. As the objective function is stochastic due to the Monte Carlo simulations, we used an optimization algorithm that minimizes the expected value of the objective function, smoothing the observed function value via a Gaussian process.

7. Limited Discussion of Related Literature
The paper would benefit from an expanded discussion of related works in particular in the motion domain ("flow parsing" by Warren & Rushton & colleagues) and the works that study the "temporal binding window", i.e. the temporal discrepancy allowed before visual and temporal signals aren't combined - closely related to "temporal context" in this paper.
A brief but explicit explanation of how the likelihood was computed—particularly how the "stochastic likelihood" is integrated across trials—would add clarity.

Following the reviewer's suggestion, we expanded our discussion in the revised manuscript to better place our findings in literature.

[Line 323] Another related factor not directly addressed in our study is the visual–vestibular temporal binding window—the time interval within which visual and vestibular cues are perceptually integrated as originating from the same event. The temporal binding window varies depending on modality, stimulus properties and individual sensitivity, ranging from as narrow as ~14 ms to as broad as ~400 ms [65]. More recent studies showed that visual motion can bias inertial heading judgments when the onset difference is ≤ 250 ms, but this influence diminishes beyond 500 ms and disappears entirely at a 1,000 ms delay [66]. In our design, the visual motion preceded self-motion by 2 seconds, placing the two cues well outside the typical temporal binding window. Thus, multisensory integration via temporal binding was unlikely to contribute to the observed effects. Instead, it is more plausible that the earlier environmental motion was subtracted from the overall retinal motion to estimate the self-motion. A subtraction of motion vectors is also performed in optic flow parsing but in the opposite way—the brain estimates environmental motion by subtracting self-motion from the overall retinal motion pattern [8,9,11,12,67–70]. While the concept of the temporal binding window remains central to multisensory integration, it was not a contributing factor in the current design and instead represents an important direction for future investigation. Accordingly, future research could broaden the study's scope by examining how synchrony interacts with optic flow parsing when only parts of the visual environment are in motion.

Reviewer #2

The authors have asked an important question here and have done an interesting experiment to address it. Most studies of cue integration have only considered how the brain combines two sources of information that are presented simultaneously. But, in real life, the onset and offset of different sources of information may not be synchronous, and there may be valuable information available in how a cue gets integrated with others depending on how it was available previously. In this case, the authors examine perception of heading in response to visual and vestibular cues, and the examine how a previously available visual cue influences perception during a period of time in which both visual and vestibular cues are available.

This is a valuable topic to investigate, and there are many things to like about the study. However, it also has some important limitations that need to be investigated and discussed further. There are also some crucial technical details missing that are important to evaluate the quality of the stimuli.

Major issues:
1) It is not clear how the authors matched the dynamics and latency of the visual and vestibular stimuli. They show both as having a roughly trapezoidal waveform that is synchronous. But any motion platform is going to have dynamics and some latency. Those dynamics will make the actual movement trajectory deviate from the command signal that is sent to move the platform. So if the authors are equating the platform command signal with their visual motion trajectory, then the end result is going to be visual and vestibular stimuli that are not actually the same. It is crucial to get this right, otherwise subjects might be able to tell that the visual motion they are seeing is not consistent with the platform motion they are feeling, which would compromise the design of the study. The authors need to explain how they addressed these issues and provide evidence that the two types of stimulation indeed have the same timing and the same temporal profile.

Thank you for the comment regarding the importance of matching the *actual* motion dynamics of the motion platform to the visual stimulus. To address this, we implemented a four-step synchronization procedure. First, both visual and vestibular stimuli were designed to follow identical trapezoidal velocity profiles, with matched refresh rates (60 Hz) to ensure temporal correspondence at the command level. Second, recognizing that the platform's physical response may lag behind its command due to inherent mechanical and control system dynamics, we empirically measured the actual onset delay using the platform's real-time motor feedback that reads from sensors (encoders) mounted on the output shafts of each motor, capturing the latency between command issuance and the motor movement. This revealed a consistent 5-frame difference, corresponding to an onset delay of approximately 83.33 ms at a 60 Hz sampling rate. Third, this empirically derived delay was applied to the visual stimulus to align the perceived onset timing with the platform's physical motion. Finally, we conducted a perceptual calibration by presenting a moving dot (mirroring the platform trajectory) on the visual display, aligned with a central physical reference marker. Finally, to confirm trajectory alignment, we presented a moving dot on the visual display (mirroring the platform motion) and aligned it with a physical reference marker placed on the aperture mounted to the motion platform, ensuring that both visual and vestibular stimuli appeared synchronous from the participant's viewpoint. This systematic procedure ensured that both modalities were temporally aligned in both command and actual motion, preserving the ecological validity of the multisensory stimulus and avoiding perceptual discrepancies.

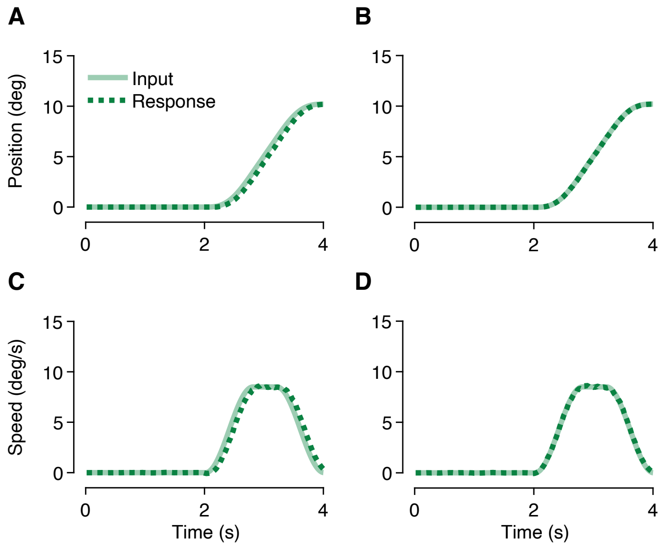

Fig. S1. Motion platform synchronization before and after delay compensation. (**A**,**B**) Position profiles of the commanded trajectory (light green) and measured response (dark green) before (**A**) and after (**B**) applying an 83 ms delay compensation. (**C**,**D**) Corresponding speed profiles by differentiating the position data and smoothing with a Savitzky-Golay filter (window size: 450 ms).

[S1 Appendix] To ensure precise temporal alignment between the visual and vestibular stimuli, we implemented a synchronization procedure. The results of this procedure are shown in **Fig. S1**, which displays the commanded and measured motion profiles before and after delay compensation.

Both the input command to the motion platform and the resulting physical displacement are represented in terms of position. While the input was specified in millimeters, the actual platform movement was monitored using encoder data expressed in hardware-specific counts. These encoder counts, though not directly in millimeters, are linearly proportional to the motor shaft rotation and thus to the crank arm angle driving the platform's translation. Because of the fixed mechanical linkage, this proportionality ensures that encoder counts reliably track changes in linear displacement, allowing us to compare commanded and actual motion trajectories with consistent temporal precision. The precise conversion factor between encoder counts and millimeters was handled by the platform's control system and was not required for the delay analysis, which relied on relative timing and linearity between command and response trajectories. By comparing the commanded trajectory (input) with the measured encoder-derived trajectory (response) shown in **Fig. S1A**, we observed a consistent delay in the platform's physical response relative to the command. Specifically, the response onset lagged behind the input by approximately 5 frames, or 83 ms at a 60 Hz sampling rate.

To correct for this measured delay, the onset of the visual stimulus was shifted forward by 5 frames (83 ms), thereby aligning its perceived motion onset with the actual movement of the platform. After compensation, the alignment between the commanded and measured profiles improved significantly (**Fig. S1B**), demonstrating that the visual and vestibular stimuli were synchronized in both time and trajectory shape.

As an additional qualitative verification, a moving dot mimicking the platform motion was displayed on the screen and aligned with a physical reference marker mounted on the platform. From the participant's viewpoint, the moving dot and the physical marker moved in unison, confirming perceptual synchrony between visual and vestibular motion cues.

Although our calibration procedure cannot remove all the discrepancy between input and response due to the inherent noise in the system, the difference between input and output speed profiles after our calibration procedure (**Fig. S1D**) falls well within the known temporal window of integration for visual and vestibular signals. For instance, Rodriguez & Crane [S1] reported that visual motion influences inertial heading perception when intermodal timing differences are within 250 ms, indicating that our system remains well within the perceptual threshold for the natural multisensory integration.

2) The stimulus situation that the authors have concocted is naturalistic in some ways but unnatural in others. Specifically, all of the visual motion that subjects see in their visual field is unidirectional and caused by environmental motion. Apart from a situation in which a moving object is so large and so nearby that it takes up the entire visual field, this is something that never happens in real life. In real life, the visual motion field includes some components that associated with self-motion (i.e., stationary background elements) and some that reflect both self-motion and object (environmental) motion (e.g., the example that the authors themselves give in the first paragraph of the Introduction). In those situations, there are other mechanisms, such as optic flow parsing, that also contribute to how we perceive object motion and self-motion. The probabilities that subjects apply to the different motion inputs in this experiment might therefore be shaped by this design, or their actual priors (formed from natural experience) might not be very compatible with the stimulus situation that is depicted. I'm not suggesting that the authors need to re-do their experiments using a more naturalistic stimulus, but I do think that it is necessary that they discuss these limitations at some length and provide some appropriate caveats related to how their findings might generalize.

We agree with the reviewer's point that the stimulus situation used in our experiment is not representative of typical everyday visual experiences, where the visual field is composed mostly of stationary objects and backgrounds, with only occasional moving elements. In such naturalistic settings, as the reviewer noted, optic flow parsing becomes an important consideration. However, our choice of stimulus was not to emulate the natural environment but to investigate a scenario designed to maximize the effect size of the targeting mechanism—specifically, the effects of synchrony. While this may represent an extreme case, we believe it offers the clearest conditions under which the targeted mechanism can be revealed. It is common practice in vision science to use unnatural, extreme visual stimuli to reveal an underlying perceptual mechanism clearly. Importantly, we do not believe that this choice undermines the validity of our findings; rather, it highlights the mechanism in its most discernible form. Furthermore, we would like to point out that there are real-world situations in which the entire visible area is in motion. In addition to the example we introduced in the Introduction—where a large bus fills most of the visual field—observers inside an airplane or a train often view the outside world through an aperture that can be completely filled by another airplane or train. The stimulus situation in our experiment resembles these cases. We acknowledge that extending this investigation to more naturalistic conditions—such as scenarios involving optic flow parsing, particularly when moving objects are presented earlier—would be a valuable direction for future research. We addressed these limitations in the Discussion, as follows:

[Line 317] The stimulus used in our study—in which the entire visible environment moves uniformly—is uncommon in everyday life. Aside from the example introduced in the Introduction, where a large bus fills most of the visual field, rare instances include situations where observers inside a train view the outside world through an aperture entirely filled by the motion of another train. In typical daily visual experiences, the visual field consists mostly of stationary objects and backgrounds, with only occasional moving elements. This simplification of the experimental setup is a limitation of the current study; however, it also enables a clearer identification of the targeted mechanism: the effects of synchrony between visual and vestibular signals.

3) The authors fit the behavioral data with a causal inference model. In this model, the two causal interpretations are that environmental motion is constant before and during self-motion (C=1) or that the environmental motion changes in the time period before vs. during self-motion (C=2). There are a few aspects of this that are awkward. A) It is a bit strange to use this type of model when the subjects are never asked to make this causal judgment. Hence, the data may not really constrain fits of this type of model very well. This is a major limitation that needs to be at least discussed explicitly.

We would like to first point out that it is still possible for observers to perform causal inference even when they are not asked to make causality judgments. That said, while directly asking observers to make causality judgments could help constraining the model fit, we were concerned that it might induce observers to perform causal inference computations that they might not have done otherwise. Therefore, we believe a stricter test for causal inference in perception can be done without asking observers to report causality judgments.

A bit more trivial concern was that, if we were to ask observers to report both their perceived heading direction and causality judgement (i.e., dual-report task) or let them know which of those two they have to report only after the stimulus presentation, their heading estimates can be contaminated by a confirmation bias such that the observers tend to under or overweight what they saw before self-motion in order to be consistent with their *conscious* judgment of the causal structure. If we were to instead ask observers to report heading estimates and causality judgements in different trials, blocks or sessions, they might focus more on inertial motion stimuli when they are asked to report their perceived heading direction and on visual motion stimuli when they are asked to report their causality judgement about whether environmental motion has remained constant, leading to different visual and vestibular uncertainty depending on the task and failing to constrain the model parameters.

Numerous studies on causal inference (from different groups using different tasks) did not ask observers to explicitly report their causality judgments (e.g., Knill, 2007; Dokka et al., 2010; Rohe & Noppeney, 2015; de Winkel et al. 2017; de Winkel et al., 2018; Beierholm et al., 2020; Hong et al., 2021; Badde et al., 2023; Li et al., 2025), including one of the papers the reviewer cited elsewhere (Shivkumar et al., 2025). Following the existing work in literature, we chose to not include the causality judgement task. Nevertheless, we believe that our model provides a good fit to the data even without causality judgements, as evidenced by high R^2 values that we now report in response to Reviewer #1's comment (**Line 183**), and by model comparison results that we included in the revised manuscript (please see below for our response to comment #4).

[Line 183] The fitting results showed that the CCI model provides an excellent fit to the psychophysical data, with average R^2 and SEM of 0.714 ± 0.017.

[Line 295] One advantage of modeling the causal structure as a random variable is that it becomes straightforward to explain how observers make a causality judgment [23]. While we did not collect explicit causality judgments in this study, such data may help constrain the model fit.

B) The authors assume that the subjects have a prior probability on C=1 vs C=2 that is constant and matched to the stimulus statistics. But there is a good chance that this might not be the case. Previous studies have shown that different observers can have quite different prior probabilities over differ possible motion causes (e.g., Shivkumar et al. 2024). So this is a weakness that needs to be called out.

We thank the reviewer for pointing this out. We agree that observers may have priors different from the actual stimulus statistics, with a possibility of a meaningful inter-individual variability. To address this point, we now model the prior probability of $C = 1$ as a free parameter (**Line 432**).

[Line 432] We assume that C follows a binomial distribution with $p(C = 1) = p_{\text{constant}}$, which represents the prior probability that the environmental motion remains constant before and during self-motion. We treat p_{constant} as a free parameter, as it has been shown to be stable within individual observers but not necessarily tied to the statistics of the task [74], at least without a prolonged exposure [75].

C) The authors' prediction under the "momentary strategy" seems to assume that the environment is moving before self-motion and that it abruptly becomes stationary during self-motion. In reality, it seems possible that the subjects perceive the background to be moving in the world at one speed before self-motion and at another speed during self-motion. This percept could be fit by the authors model, but it doesn't mean that it is a good model for what subjects experience. So this should be discussed. And if there is any temporal mismatch in the visual and vestibular stimuli, that would greatly complicate the interpretation of this. It would have been useful for the authors to at least debrief their subjects about their interpretation of the stimuli.

We would like to start by reassuring that the observers were presented with visual and vestibular stimuli that are precisely in sync with each other (please see our response to comment #1). Thus, what the observers see gradually changed as they themselves gradually started moving.

That said, we believe that in this study we are actually modeling what the reviewer described. It is totally possible, as the reviewer described, that the observers perceive the environment to be in motion at one speed before self-motion and at another speed during self-motion. This is exactly the situation our model assumes in the $C = 2$ scenario. In this situation, what the observers saw before self-motion no longer conveys information about environmental motion during self-motion (but see below). Trying to isolate environmental motion from retinal motion during self-motion by factoring out vestibular signals will be helpful for estimating the environmental motion. But it is not helpful for estimating the heading direction, as it becomes circular if the observers subtract the resulting estimate of environmental motion from retinal motion in order to estimate the heading direction. Thus, the only reasonable thing the observers can do is to rely on their prior belief that the environment tends to be stationary. As a result, the observers attribute retinal motion entirely to self-motion. Note that in this case the uncertainty associated with this visual estimate of self-motion becomes relatively high, summing up both prior and sensory uncertainty. This is a reasonable estimation process both functionally and mathematically–if one does not have any data, one has to rely on assumptions. However, we do agree that this part was not very clear in the original manuscript. We now carefully edited the manuscript to better explain the problem the observers face in our task and the optimal solution to the problem (**Lines 506** and **514**).

[Line 506] A key point here is that x_{vis} depends both on s_{self} and s_{env}, while x_{vis0} depends only on s_{env}. Hence, the observer can infer s_{env} from x_{vis0}, which can be then used to isolate s_{self} from x_{vis}.

[Line 514] This time, x_{vis} still depends on both s_{self} and s_{env}, but there is no other sensory information about s_{env}, leaving the observer to rely solely on the prior $p(s_{\text{env}})$ to infer s_{env} in order to isolate s_{self} from x_{vis}.

It is worthwhile to note that our modeling results are consistent with a previous study that used a causal inference model to explain heading perception in the presence of object motion (Dokka et al., 2019). When a moving observer views an object that is moving independently, retinal motion of the object depends on not only object motion but also self-motion. Therefore, when estimating the heading direction, the observer has no choice but to infer the object motion based solely on the prior belief about object motion. As a result, the observer attributes retinal motion of object motion entirely to self-motion, but uncertainty associated with this object motion-based estimate of self-motion becomes exceedingly high, summing up both prior uncertainty and sensory uncertainty. In the revised manuscript, we discuss this similarity as well (**Line 273**).

[Line 273] Our causal inference model indicates that observers use their prior belief about environmental motion to estimate self-motion. Although its specific functional form differs across studies [25,36,37], it is generally believed that human observers use a speed prior centered on zero [26]. In our model, when environmental motion has remained constant before and during self-motion, observers use the retinal motion signal collected before self-motion to parse out the environmental motion from the retinal motion signal collected during self-motion. On the other hand, when environmental motion has changed, the observers are left with no other information about environmental motion except for their prior belief. Therefore, observers assume, with much uncertainty, that the environment is stationary, thereby attributing retinal motion entirely to self-motion. Our model is consistent with previous work that showed how the slow-speed prior influences heading perception in the presence of object motion [7]. Similar to our model, their model relies on the prior belief that objects in the environment tend to be stationary, attributing retinal motion of the object entirely to self-motion, but with increased uncertainty.

Another possibility is that observers assume that environmental motion before and during self-motion is not independent but correlated. This way, they don't have to rely solely on the assumption that the environment tends to be stationary. They can instead infer environmental motion during self-motion from what they saw before self-motion by exploiting the correlation structure of environmental motion. Note that even with this additional source of information, the computational process is the same with what we described above. Indeed, when the correlation is set to zero, the computation reduces to exactly what we described above. In the revised manuscript, we included this model and showed that our Contextual Causal Inference model provides better fit to the data both in terms of model comparison metric (i.e., AIC) and a unique prediction of a diagnostic pattern in the data (please see below for our response to comment #4).

4) Just because the predictions of a Bayesian model fit the data doesn't demonstrate that subjects are performing a Bayesian computation. In this study, since the authors did not vary the reliability of the different motion cues, it is not clear to me that one could reject other simpler models. Given that the data and prediction curves in Fig. 2A are just linear functions of speed, it seems like other models that do a weighted sum of the cues might also fit the data well. Can the authors demonstrate that a Bayesian model is necessary in this context? Can they demonstrate that it is possible to rule out simpler models?

We thank the reviewer for the helpful comments. In the revised manuscript, we addressed them in two distinct and complementary ways: 1) model fitting and model comparison of multiple alternative models, and 2) demonstration of a diagnostic pattern of causal inference (and not cue combination with a fixed weight).

We thank the reviewer for the valuable comments. In the revised manuscript, we performed two lines of analyses to address the reviewer's comments: 1) model fitting and model comparison of alternative models that have been considered in the previous studies, and 2) demonstration of a qualitative pattern of causal inference (and not cue combination with a fixed weight).

As suggested by the reviewer, we built a Fixed Weight (Fix) model that produces a heading estimate as a linear integration of sensory signals with fixed weights.

[Line 566]

$$\hat{s}_{\mathrm{self}} = \alpha_{\mathrm{vest}} x_{\mathrm{vest}} + \alpha_{\mathrm{mom}} x_{\mathrm{vis}} + \alpha_{\mathrm{cont}} (x_{\mathrm{vis}} - x_{\mathrm{vis0}}) \tag{35}$$

We also considered other alternative models such as conventional causal inference model in literature (Momentary Causal Inference model; MCI) that does not consider environmental motion presented ahead of self-motion, Integration (Int) model that always assumes that the environmental motion is constant before and during self-motion, Segregation (Seg) model that always assumes that the environmental motion has changed, and Covariance (Cov) model that assumes environmental motion before and during self-motion covaries and therefore performs a linear integration of sensory signals.

We found that unlike the Contextual Causal Inference model, alternative models that does not consider all available sensory information (i.e., vestibular, momentary vision, contextual vision) cannot reproduce the qualitative patterns of the heading bias (**Fig. 4B**) with linear regression coefficients significantly different from those of human observers (**Fig. 4C**). We quantitatively compared goodness-of-fit of models using AIC (**Fig. 4A**). All results confirm that the Contextual Causal Inference model outperforms other simpler models.

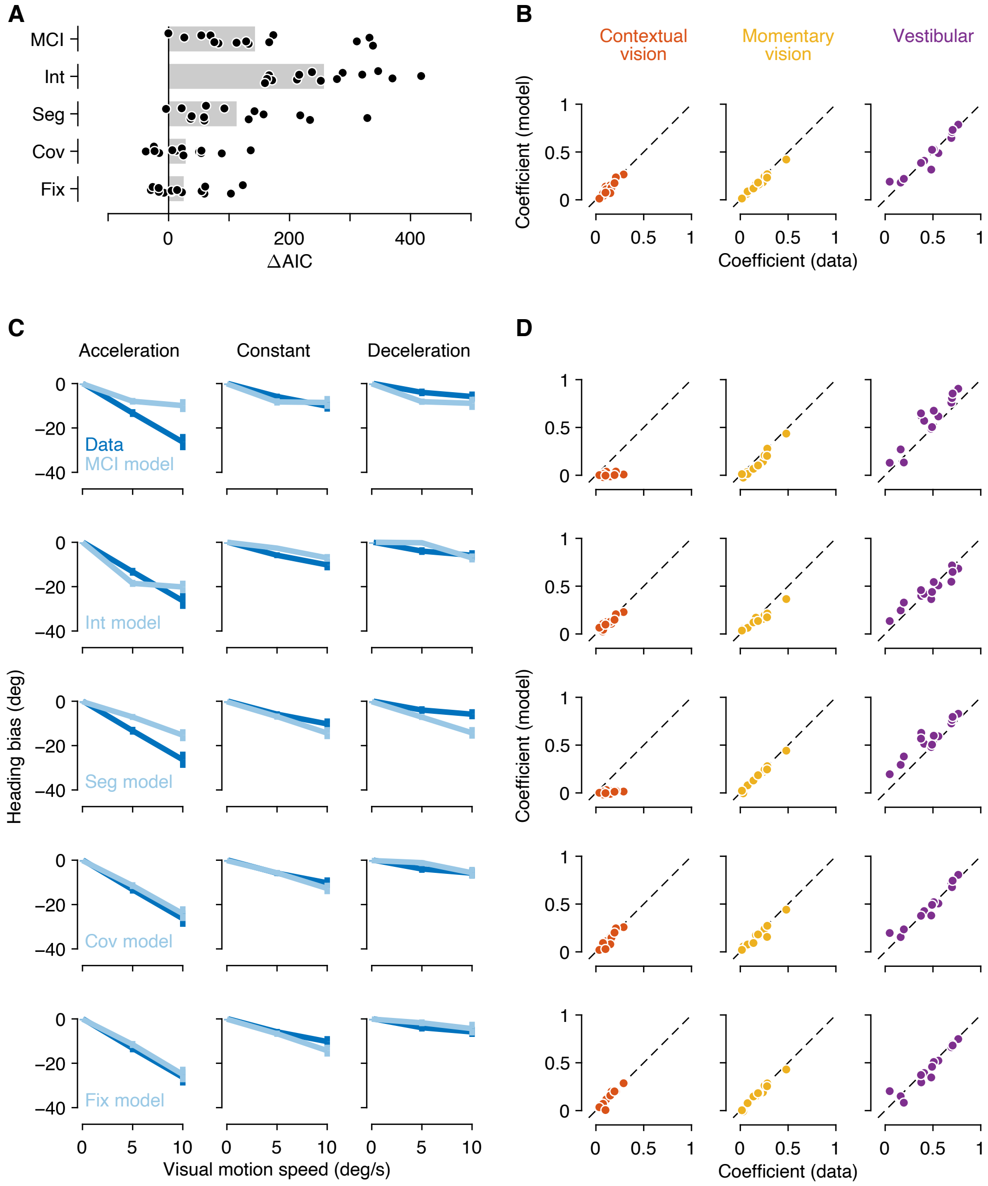

Fig. 4. Alternative models. (**A**) Comparison of goodness-of-fit among observer models. Small dots represent individual observers' difference in Akaike information criterion (AIC) values between each model and the CCI model, and gray bars represent the average. Positive values indicate a worse fit than the CCI model. (**B**) Comparison of linear regression coefficients between human and CCI model observers. (**C**) Same as in **Fig. 3A** but for alternative models. (**D**) Same as in **B** but for alternative models.

We also asked whether observers performed a linear integration of sensory signals with fixed weights, without inferring the probability of each scenario. Our Causal Inference model indicates that observers evaluate the degree to which distinct sensory cues are different from one another (with respect to the sensory uncertainty associated with each cue) and use it as a weight on the heading estimates associated with the corresponding scenario. Therefore, the observers would use a larger weight on heading estimates associated with the constant environmental motion (i.e., $C = 1$) scenario when the visual motion stimuli presented before and during self-motion are actually the same, and use a smaller weight when they are actually different. To test this, we allowed the weight on the heading estimate associated with the $C = 1$ scenario to vary for trials where the environmental motion was actually same or different before and during self-motion. We found that, consistent with the causal inference prediction, human

observers adaptively adjusted their weights according to whether the environmental motion was constant (**Fig. 5**).

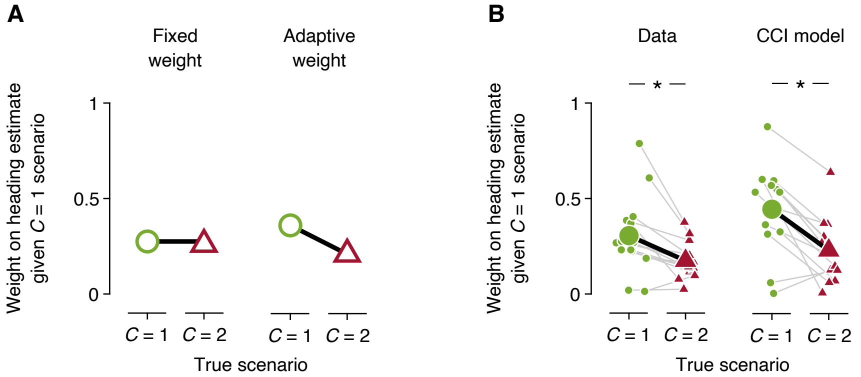

Fig. 5. Adaptive weight as a signature of causal inference. (**A**) Prediction about weights on the heading estimate associated with constant motion in the environment. For the linear sensory integration with fixed weights (left), the weights would be the same. For the nonlinear integration with adaptive weights (right), the weights would be larger when the environmental motion was constant (i.e., $C = 1$). (**B**) Weights on the heading estimate associated with constant motion in the environment for human (left) and the CCI model (right) observers. Small markers connected by gray lines represent weights for individual observers, and big markers connected by a black line represent the corresponding averages. $^*P < 0.05$.

Finally, we acknowledge that an adaptive weight does not necessarily mean observers performed causal inference in a Bayes-optimal manner. It is also possible that observers did it suboptimally. To test for this possibility, we fit a Heuristic model which compares cue disparities without taking into account the cue reliability and a Winner-Take-All model that performs a model selection. We found that both models provide a significantly worse fit than our causal inference model for all observers' data.

[Line 242] The results that the observers performed a contextual causal inference does not necessarily mean they did it optimally. To test this, we fit two variants of causal inference model: a Heuristic model that performs the causal inference without taking into account sensory uncertainty and a Winner-Take-All model that commits entirely to the more probable scenario without taking into account the less probable one. Both the Heuristic model (64.7 [39.5 93.1]) and the Winner-Take-All model (39.7 [20.3 60.5]) showed a significantly worse fit than the CCI model.

Other specific issues:
5) Line 172: "The causal inference framework has been wildly employed…" I presume the authors meant "widely employed"?

We thank the reviewer for catching this out. The typo has been fixed.

6) Given that the visual stimulus is not a typical type of optic flow field, it is important that the authors include a video that shows examples of the stimuli.

We included three example videos of the stimuli (**S1–3 Movies**).

7) Lines 273-275: This part about symmetry was hard to follow. Please rewrite and clarify.

Following the reviewer's suggestion, we added detailed explanations of how we computed the heading bias shown in **Fig. 3B**.

[Line 406] To assess the observers' perceptual performance, we calculated their heading bias as the average angular difference between the perceived heading direction reported by the observers and the true heading direction, with errors realigned such that positive deviations are in the direction of the visual motion stimuli. Thus, a negative heading bias indicates that the heading estimates are biased in the direction opposite to the visual motion stimuli in the frontal plane. Our analysis leveraged the symmetry of the heading bias by collapsing the bias onto one side of the graph. This was achieved by mirroring each observer's heading bias in the origin, expressing it as a function of the speed, not velocity, of visual motion stimuli. That is, we flipped the sign of the heading bias on the trials with the leftward (i.e., negative) visual motion, and plotted the heading bias on all trials as a function of unsigned visual motion velocity (i.e., visual motion speed).

8) Lines 278-282: The explanation of the momentary and contextual strategies, both here and in the main text, was not very clearly explained. I had to read these sections several times to get it. Perhaps a diagram that illustrates these strategies would aid some text revisions. Or it may be useful to give equations to make the predictions of these strategies more explicit.

We thank the reviewer for the thoughtful comments. We agree that the explanation about the linear regression analysis was not very clear in the original manuscript. Following the reviewer's suggestion, we now included a diagram illustrating the conceptual framework (**Fig. 1A**) and the three strategies (**Fig. 3A**) and an equation for the linear regression analysis (**Line 420**).

[Line 420] To quantitatively assess contributions of each source of information, we fit a linear regression model with the observers' heading estimate as a dependent variable and the predicted heading bias for each source of information as independent variables:

$$\hat{s}_{\mathrm{self}} = \beta_0 + \beta_1 s_{\mathrm{self}} + \beta_2 (s_{\mathrm{self}} - s_{\mathrm{env}}) + \beta_3 (s_{\mathrm{self}} - s_{\mathrm{env}} + s_{\mathrm{env0}}) \quad (6)$$

where s_{self} represents self-motion and s_{env} and s_{env0} represent environmental motion before and during self-motion, respectively (see also below).

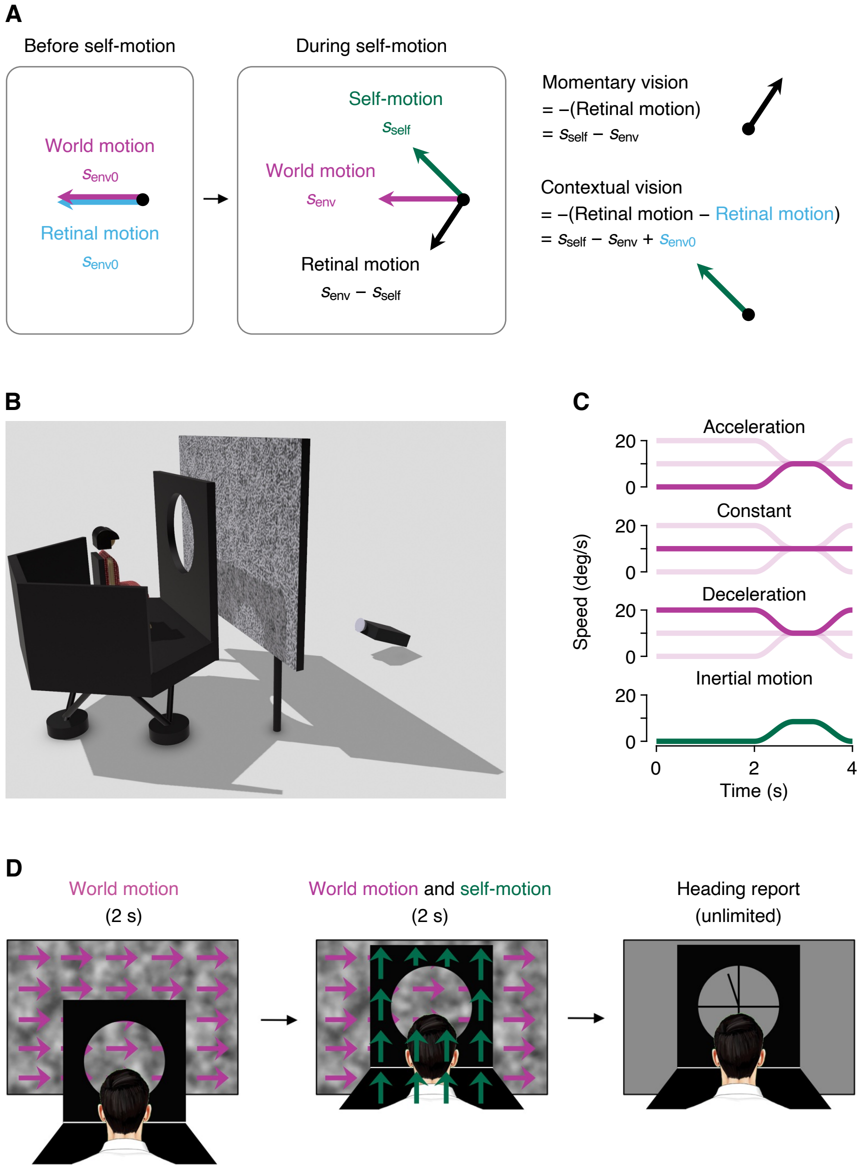

Fig. 1. Experimental paradigm. (**A**) Conceptual framework. Before an observer begins to move, retinal motion is solely due to motion in the world (left). As the observer starts moving while the world motion continues, retinal motion reflects the vector subtraction of world motion and self-motion (center). If the observer interprets retinal motion at face value, their heading estimate will be opposite to the retinal motion (top-right). If the observer has a good reason to believe that world motion has remained constant before and during self-motion, they can estimate their heading by subtracting retinal motion before self-motion from retinal motion during self-motion (bottom-right). (**B**) Experimental setup. Observers sat on a motion platform and viewed visual motion stimuli on a rear-projection screen through a circular aperture. The screen was positioned outside the motion platform, while the aperture was mounted on and moved together with the motion platform. (**C**) Example velocity profiles for visual motion stimuli (magenta) across three conditions (i.e., Acceleration, Constant and Deceleration) and for inertial motion stimuli (green). For comparison, the velocity profiles of the other two visual motion conditions are shown in light magenta. (**D**) Experimental task. Observers were shown leftward or rightward visual motion (indicated by magenta arrows, not shown in the experiment) for two seconds. After the two seconds, as visual motion continued, observers were passively moved along one of ten directions in the frontal plane ranging from –45° to 45° relative to vertically upward (indicated by green arrows, also not shown in the experiment). Following the synchronized offset of both visual and inertial motion stimuli, observers reported their perceived heading direction by adjusting a probe on the screen using a computer mouse.

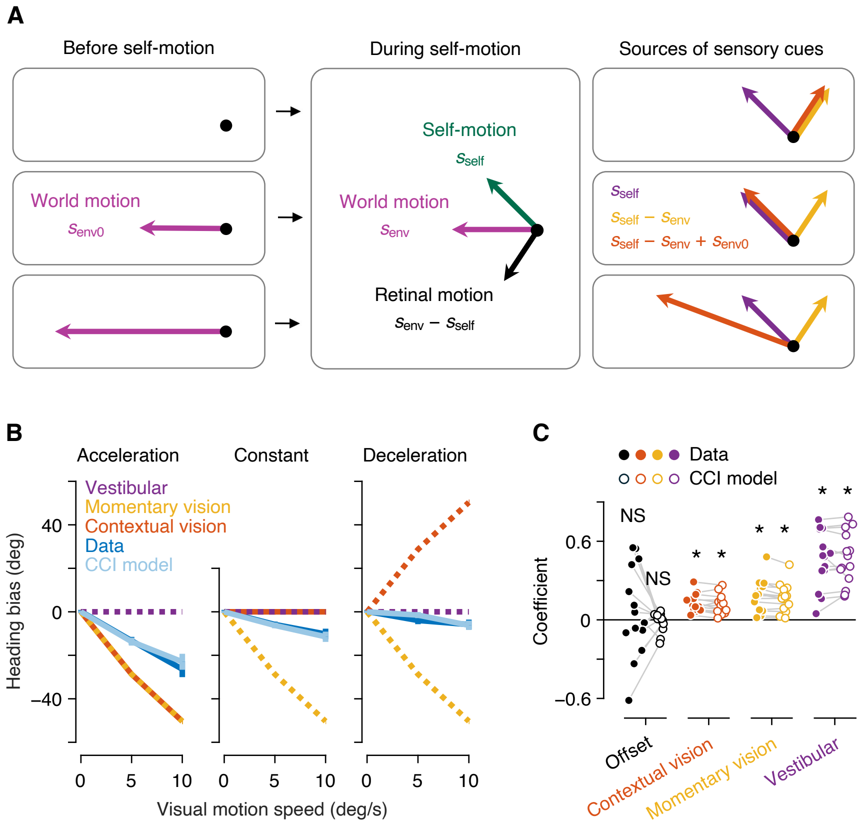

Fig. 3. Heading bias depends on environmental motion presented before self-motion. (A) Schematic illustration of three sources of sensory cues for Acceleration (top), Constant (middle) and Deceleration (bottom) conditions. (**B**) Heading bias as a function of visual motion speed across three conditions (i.e., Acceleration, Constant and Deceleration). A negative heading bias indicates that observers' heading estimates are biased in the direction opposite to the visual motion stimuli. Dotted lines represent the predicted heading bias when using vestibular (purple), momentary vision (yellow) and contextual vision (red) strategies exclusively. Solid lines represent human (dark blue) and Contextual Causal Inference (CCI) model (light blue) observers' heading bias. Error bars represent the standard error of the mean. (**C**) Linear regression coefficients for human (filled dots) and model (open dots) observers. $*P < 0.05$, NS $P > 0.05$.

9) The authors' model includes a zero-mean Gaussian prior on environmental motion. While such a slow speed prior has been used by others, recent studies (e.g., Shivkumar et al. 2024; Penaloza et al. 2024) have argued that this is not a good prior for object motion. Most objects in a scene are stationary and have exactly zero speed. Those that are moving may be drawn from a zero-mean Gaussian prior. Thus, others have argued for the use of a bipartite prior (e.g., a spike at zero speed combined with a Gaussian) to model object motion in the world. The authors should at least discuss this issue, acknowledge the alternatives, and describe why they favor their model.

In the revised manuscript, we acknowledged that the speed prior has been proposed to take functional forms different from Gaussian, both when we first define the slow speed prior (**Line 437**) and when we discuss the influence of the speed prior (**Line 273**; reproduced in our response to comment #3C).

[Line 437] Motivated by the rich literature on the slow-speed prior [25–27,81], we assume that s_{self} follows a zero-mean Gaussian prior distribution $\mathcal{N}(0, \sigma_{self}^2)$, and s_{env0} and s_{env} follow a zero-mean

Gaussian prior distribution $\mathcal{N}(0, \sigma_{\text{env}}^2)$. If the environmental motion before and during self-motion is constant (i.e., $C = 1$), s_{env0} is drawn from the prior, and s_{env} takes the same value. If the motion is independent (i.e., $C = 2$), both s_{env0} and s_{env} are independently drawn from the same prior. Note that while many previous models have assumed Gaussian priors [7,26,82–84] or Gaussian process priors such as Ornstein-Uhlenbeck process [28–32] for analytical tractability, the specific functional form of the slow-speed prior has been debated, with other models assuming a power-law function [25,27,81,85,86] or mixture priors with a point-mass at zero [36,37]. The structure of our model is compatible with such alternatives, though extensions may require additional free parameters and/or numerical rather than analytic solutions even in early stages of the derivation [14].

10) It would be useful to have a clear statement that describes how many free parameters there are in the Bayesian model that is fit to data. One can count them up if they try, but it would be useful to make this easy on the reader. Also, were there any bounds or other constraints on the model parameters in fitting?

In response to the reviewer's comment, we added detailed explanations of model fitting (starting from **Line 617**). Following the reviewer's previous comments, we also fit other alternative models in the revised manuscript, and some of them were not well constrained by the data. Therefore, we further constrained the model fitting using log-normal priors on the model parameters, with 95% of prior mass allocated between 1/7 and 7 times the median. We confirmed that the log-likelihood of maximum-a-posteriori parameter estimates is comparable with that of maximum-likelihood estimates. All the model comparison results were the same regardless of using maximum-likelihood vs. maximum-a-posteriori parameter estimates. Please note that this constraint was made mostly because of the unstable parameters of the alternative models, presumably due to their inability to naturally explain the structure of the data, and our causal inference model did not suffer from unstable parameter fits. We included all these details in the manuscript.

Reviewer #3

In the current study, the authors examined effect of prior visual motion information on vestibular-based self-motion direction judgment. The main finding is that when there is a matching temporal dynamics of visual prior and vestibular signals, the visual would bias the vestibular-based heading judgment. When there is a difference, however, the influence is small. The authors used a Bayesian causal inference model to explain this result. I found this research and finding is very interesting. The conclusion makes sense. The paper is clearly written.

We thank the reviewer for the positive evaluation.

The only unclear part for me is about the three visual conditions with different prior temporal dynamics. Are the three stimulus condition randomly interleaved in one session? It is not clearly described in the method. If they are interleaved, the authors may examine the inter-trial effect, that is, the prior trial on the current judgment.

We apologize for the ambiguity in the Methods section. To clarify, the three visual stimulus conditions with different prior temporal dynamics were randomly interleaved within a single session. We will revise the manuscript to explicitly state this in the revised Methods section.

[Line 400] There was a total of 150 distinct stimulus conditions (5 visual motion velocities × 10 inertial motion directions × 3 visual motion conditions), and each condition was repeated five times, resulting in a total of 750 trials. The three visual motion conditions, each defined by different prior temporal dynamics, were randomly interleaved within each session to prevent predictability and ensure proper counterbalancing. The experiment was conducted over two sessions, with each session consisting of 15 blocks of 25 trials each.

The other question is that as my understanding, the visual motion is always lateral (left or rightward), while the vestibular motion is mainly upward, which is quite different from the visual in terms of motion direction. So why the subjects cannot exclude the visual stimulus (even identical temporal dynamics), due to a large difference in the direction. If there are trials in which the two modality inputs show similar directions, then this is understandable. Otherwise I may have misunderstood their methods.

We thank the reviewer for the opportunity to clarify the stimulus setup. In our experiment, the visual stimulus was presented on a screen located outside the motion platform, and observers viewed it through an aperture. As a result, the visual stimulus naturally moved downward in sync with the platform's motion, while horizontal visual motion was superimposed on the motion induced by the body movement. Consequently, the direction of retinal motion reflected the combined effects of both body-induced motion and the actual visual motion displayed on the screen. We clarified the description of the stimulus setup as follows:

[Line 356] The distance between the screen and the aperture was 43 cm, and the distance between the aperture and the observer was 60 cm, resulting in a visual field with a radius of 25°. To prevent access to extraneous visual cues outside the intended visual field, opaque side panels were integrated around the headrest. These panels acted as peripheral occluders, ensuring that participants could only see the visual stimuli presented through the aperture and were shielded from any surrounding environmental cues.

The other improvements may be that there could potentially be more operations on the stimulus parameters. For example, reliability of the vestibular cue could be introduced to examine the weight effect. In another example, more stimulus could be introduced between the three visual conditions (e.g. up to 5), to see a more smooth curve of performance under causal inference model. However, I understand these may increase the number of trials, and thus may be considered in future experiments.

We thank the reviewer for the insights. As the reviewer noted, we had to balance ourselves between having sufficient repetitions and having additional stimulus conditions. This was especially challenging, because our experimental design entailed particularly long visual and inertial motion stimuli (4 seconds) as well as long inter-trial interval (~3 seconds) during which the motion platform moved back to its initial location. Therefore, we decided to have a single level of cue reliability, i.e., strong visual cues to reliably test the effect of visual motion presented before self-motion. As for the weight effect (and the performance of the causal inference model), weights in the causal inference model depend not only on cue reliability, but also on cue disparity. In the revised manuscript, we tested whether observers adaptively adjusted the weights depending on whether environmental motion before and during self-motion was constant (**Fig. 5**)

We also tested whether observers relied on heuristics to arbitrate between integration and segregation of multisensory cues, without taking into account the cue reliability. Specifically, we fit a Heuristic model that compares cue disparity without taking into account the cue reliability and found that it provides a significantly worse fit than our causal inference model for all observers' data (**Line 232**).

[Line 242] The results that the observers performed a contextual causal inference does not necessarily mean they did it optimally. To test this, we fit a two suboptimal causal inference model: a Heuristic model that performs the causal inference without taking into account sensory uncertainty and a Winner-Take-All model that commits entirely to the more probable interpretation without taking into account the less probable one. Both the Heuristic model (64.7 [39.5 93.1]) and the Winner-Take-All model (39.7 [20.3 60.5]) showed a significantly worse fit than the CCI model.

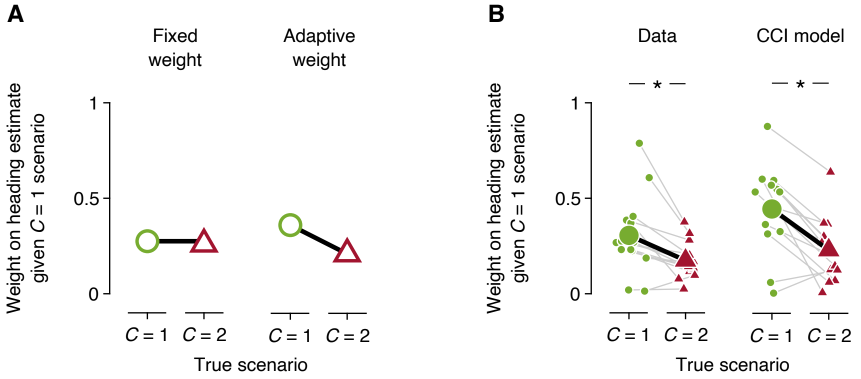

Fig. 5. Adaptive weight as a signature of causal inference. (**A**) Prediction about weights on the heading estimate associated with constant motion in the environment. For the linear sensory integration with fixed weights (left), the weights would be the same. For the nonlinear integration with adaptive weights (right), the weights would be larger when the environmental motion was constant (i.e., $C = 1$). (**B**) Weights on the heading estimate associated with constant motion in the environment for human (left) and the CCI model (right) observers. Small markers connected by gray lines represent weights for individual observers, and big markers connected by a black line represent the corresponding averages. $^*P < 0.05$.

References

Badde, S., Landy, M. S., & Adams, W. J. (2023). Multisensory causal inference is feature-specific, not object-based. *Philosophical Transactions of the Royal Society B*, *378*(1886), 20220345.

Beierholm, U., Rohe, T., Ferrari, A., Stegle, O., & Noppeney, U. (2020). Using the past to estimate sensory uncertainty. *eLife*, *9*, e54172.

de Winkel, K. N., Katliar, M., & Bülthoff, H. H. (2017). Causal inference in multisensory heading estimation. *PLoS One*, *12*(1), e0169676.

de Winkel, K. N., Katliar, M., Diers, D., & Bülthoff, H. H. (2018). Causal inference in the perception of verticality. *Scientific Reports*, *8*(1), 5483.

Dokka, K., Kenyon, R. V., Keshner, E. A., & Kording, K. P. (2010). Self versus environment motion in postural control. *PLoS Computational Biology*, *6*(2), e1000680.

Dokka, K., Park, H., Jansen, M., DeAngelis, G. C., & Angelaki, D. E. (2019). Causal inference accounts for heading perception in the presence of object motion. *Proceedings of the National Academy of Sciences*, *116*(18), 9060-9065.

Girshick, A. R., Landy, M. S., & Simoncelli, E. P. (2011). Cardinal rules: visual orientation perception reflects knowledge of environmental statistics. *Nature Neuroscience*, *14*(7), 926-932.

Hong, F., Badde, S., & Landy, M. S. (2021). Causal inference regulates audiovisual spatial recalibration via its influence on audiovisual perception. *PLoS Computational Biology*, *17*(11), e1008877.

Jazayeri, M., & Shadlen, M. N. (2010). Temporal context calibrates interval timing. *Nature Neuroscience*, *13*(8), 1020-1026.

Knill, D. C. (2007). Robust cue integration: A Bayesian model and evidence from cue-conflict studies with stereoscopic and figure cues to slant. *Journal of Vision*, 7(7), 5-5.

Körding, K. P., & Wolpert, D. M. (2004). The loss function of sensorimotor learning. *Proceedings of the National Academy of Sciences*, *101*(26), 9839-9842.

Li, L., Hong, F., Badde, S., & Landy, M. S. (2025) Precision-based causal inference modulates audiovisual temporal recalibration *eLife* 13:RP97765.

Rohe, T., & Noppeney, U. (2015). Cortical hierarchies perform Bayesian causal inference in multisensory perception. *PLoS Biology*, *13*(2), e1002073.

Shivkumar, S., DeAngelis, G. C., & Haefner, R. M. (2025). Hierarchical motion perception as causal inference. *Nature Communications*, *16*(1), 3868.

Wei, X. X., & Stocker, A. A. (2015). A Bayesian observer model constrained by efficient coding can explain 'anti-Bayesian' percepts. *Nature Neuroscience*, *18*(10), 1509-1517.

Zhu, H., Beierholm, U., & Shams, L. (2024). BCI Toolbox: An open-source python package for the Bayesian causal inference model. *PLoS Computational Biology*, *20*(7), e1011791.
