# Supplementary material for: Environmental motion presented ahead of self-motion modulates heading direction estimation
Source: PLoS Comput Biol. 2025 Oct 9;21(10):e1013571. doi: 10.1371/journal.pcbi.1013571 (PMC12527208; doi:10.1371/journal.pcbi.1013571)
Supplement: Attachment — Submitted filename: ResponseToReviewer_R2.pdf [file pcbi.1013571.s007.pdf]

We would like to thank the reviewers for their positive evaluations on the revision. We edited the manuscript in response to the comments from Reviewer 2. We also uploaded the data, analysis code, model fitting codes, and fitted parameters to OSF.

Reviewer #1

The authors have thoroughly addressed the reviewer comments and the revised manuscript is greatly improved. I have no further concerns.

We appreciate the reviewer's constructive comments in the first round. We believe the manuscript has been significantly improved thanks to the reviewers' feedback.

Reviewer #2

Overall, the authors have done a good job of responding to the previous reviews and most of my concerns have been addressed adequately. This study has some substantial limitations in addition to multiple strenghts, but I think the limitations are now acknowledged fairly.

One of my major previous concerns was that cue reliability was not varied and so the data may not strongly differentiate a Bayesian model from other types of models that involve a weighted sum of the cues. To address this, the authors have performed formal model comparisons with other models, and Figure 4 is a good addition to the manuscript. That said, I don't find the results of Fig. 4 to be as definitive as the authors apparently do. Specifically, for the Cov and Fix models, these simpler models perform better than the authors' CCI model for a substantial minority of subjects. It is true that the average delta_AIC values for these models are positive, but the mean values being positive are pretty strongly driven by a couple of subjects. And for a handful of subjects, the delta_AIC is negative indicating that the simple model is better. I think the authors need to explicitly acknowledge this and soften their conclusions about the superiority of the CCI model.

Partially mitigating the above issue is the new result of Figure 5, which does provide evidence that most subjects are changing their weights. Of course, if the authors had varied cue reliability in some systematic fashion, they could have provided a more powerful test of the superiority of their CCI model. I still see this as a missed opportunity, but I don't consider it to be a major issue that is fatal here.

Otherwise, the authors have addressed my concerns.

Following the reviewer's suggestion, we now explicitly state the number of observers whose data favors the CCI model over the Cov and Fix models, in addition to the data points in **Fig 4A** representing the individual observers' ΔAIC values.

[Line 189] We compared the performance of the CCI model with that of other alternative models (**Fig 4**). We first quantitatively compared the model performance using the Akaike information criterion (AIC) to account for differences in model complexity (**Fig 4A**), with results reported below as the mean AIC difference from the CCI model followed by a bootstrapped 95% confidence interval in brackets. We also compared human and model observers' heading biases (**Fig 4C**) and linear regression coefficients (**Fig 4D**) to demonstrate that the CCI model better accounts for the psychophysical data for the majority of the observers.

[Line 218] While the Cov model exhibited a qualitatively more similar pattern to the data than the above alternative models, it provided a significantly worse fit than the CCI model (28.3 [5.1 54.1]), with 10 out of 14 observers' data favoring the CCI model. We similarly considered a Fixed Weight (Fix) model, a rather descriptive model that computes a weighted sum of all available sensory signals with fixed weights, and obtained similar results (25.0 [2.2 49.9]), with 9 observers' data favoring the CCI model.
